# Assessment of leadership competence among medical students in Saudi Arabia using the MeLeCoS scale: a cross-sectional study

**DOI:** 10.3389/fmed.2026.1993737

**Published:** 2026-10-27

**Authors:** Jawad S. Alnajjar, Mohammed A. Almarzooq, Ali M. Al Dandan, Abdullah Almaqhawi

**Affiliations:** 1 College of Medicine, King Faisal University, Al Ahsa, Saudi Arabia; 2 Department of Family and Community Medicine, College of Medicine, King Faisal University, Al Ahsa, Saudi Arabia

**Keywords:** cross-sectional studies, leadership, medical education, medical students, professional competence, Saudi arabia, undergraduate

## Abstract

**Introduction:**

Leadership competence is increasingly recognized as an essential attribute for physicians, enabling effective teamwork, healthcare system management, and improvement of patient outcomes. Medical schools worldwide are increasingly integrating leadership training into their curricula; however, limited evidence exists regarding leadership competence among medical students in Saudi Arabia. This study aimed to assess self-perceived leadership competencies among medical students at King Faisal University and to explore demographic and academic factors associated with leadership competence.

**Methods:**

A cross-sectional study was conducted among medical students at the College of Medicine, King Faisal University, Alahsa, Saudi Arabia. Participants were selected using a proportionate stratified sampling technique to achieve representation across different academic years. Data were collected through a structured questionnaire consisting of demographic characteristics and the 37-item Medical Leadership Competency Scale (MeLeCoS), which assesses six domains of leadership competence: self-management, responsible behavior, teamwork, managing services, innovation in undergraduate education, and systemic perspectives. Descriptive statistics were used to summarize participant characteristics. Independent sample t-tests, one-way analysis of variance (ANOVA), Pearson's correlation, and multiple linear regression analyses were performed to identify factors associated with leadership competence.

**Results:**

A total of 191 medical students participated in the study (overall response rate 59.5%). The mean age of participants was 21.76 ± 1.53 years and the mean GPA was 4.32 ± 0.63. Female students constituted 59.7% of the sample. The overall mean leadership competence score was 3.72 ± 0.58, indicating a moderate to high level of perceived leadership competence. Among the MeLeCoS domains, responsible behavior and teamwork demonstrated the highest competence levels, whereas innovation in undergraduate education showed the lowest score. Female students had significantly higher leadership competence scores compared with male students (*p* = 0.001). Academic performance (GPA) was positively correlated with leadership competence (r = 0.217, *p* = 0.006). In multiple regression analysis, gender (*β* = 0.256, *p* = 0.002) and GPA (*β* = 0.201, *p* = 0.010) were identified as significant predictors of leadership competence. Medical students at King Faisal University reported moderate to high levels of self-perceived leadership competence, particularly in responsible behavior and teamwork. Gender and academic performance were significant predictors of self-perceived leadership competence.

**Discussion:**

These findings highlight the importance of incorporating structured leadership development programs within undergraduate medical education to enhance leadership capacity among future physicians.

## 1 Introduction

Leadership is increasingly recognized as a core competency in medicine because physicians are expected not only to provide safe and effective patient care, but also to coordinate teams, communicate across disciplines, contribute to service improvement, and respond to complex healthcare systems (1–4). In the medical literature, leadership has been discussed as an important professional competency for physicians and as a relevant component of undergraduate medical education (2, 3, 5). In this context, leadership is not restricted to formal administrative positions but is described in relation to everyday professional practice, collaboration, and responsibility within healthcare settings (2, 3, 5).

In undergraduate medical education, leadership development has been addressed in reports and systematic reviews that examined the teaching, structure, and evaluation of leadership training for medical students (6–8). These publications indicate that leadership has become an identifiable topic within the undergraduate curriculum and within broader discussions of professional competence in medicine (6–8). The Medical Leadership Competency Framework (MLCF) has been particularly influential in conceptualizing leadership across domains such as personal qualities, teamwork, service management, improvement, and strategic direction (9). Such frameworks provide an organized way to describe leadership-related competencies within medical training and practice (9).

In Saudi Arabia, the SaudiMEDs framework was developed to define the competencies required of medical graduates and to align medical education with national healthcare needs (10). A comparison between SaudiMEDs and CanMEDs also described similarities across major competency domains, including professionalism, communication, and teamwork (11). These domains are closely related to areas that are commonly discussed in the medical leadership literature and in competency-based approaches to medical education (9–11).

Published studies from Saudi Arabia have examined medical students’ perceptions of and attitudes toward medical leadership and management, the need for leadership courses in Saudi medical schools, and the views of medical students and interns regarding leadership development (12–14). These studies addressed students’ perspectives on the importance of leadership training, their views on formal leadership education, and barriers related to leadership development (12–14).

The transition from medical student to junior doctor represents an important stage in early professional and clinical practice (15). However, the available Saudi studies have focused on perceptions, attitudes, and self-reported educational needs related to leadership rather than on a structured, multidimensional assessment of leadership competence (12–14). Evidence from a systematic review suggests that positive relational leadership styles are associated with lower patient mortality, fewer medication errors, reduced restraint use, and lower rates of hospital-acquired infections (16). For undergraduate medical students, the Medical Leadership Competency Scale (MeLeCoS) was developed as a validated multidimensional self-assessment tool for leadership competence in healthcare (17). Using such a tool allows students’ self-perceived leadership competence to be assessed across defined, framework-based domains, rather than relying only on general attitudes toward leadership or perceived training needs (12–14, 17). However, because MeLeCoS is a self-assessment instrument, it captures perceived rather than observed leadership competence (17). Applying this multidimensional instrument in a Saudi undergraduate setting may also show which leadership domains students perceive as relatively well developed and which remain underdeveloped, and how this profile relates to existing competency frameworks such as the MLCF and SaudiMEDs (9–11). This study therefore aimed to assess self-perceived leadership competence among medical students at King Faisal University using the MeLeCoS and to identify demographic and academic factors associated with leadership competence.

## 2 Materials and methods

### 2.1 Study design and setting

A cross-sectional study was conducted among undergraduate medical students at the College of Medicine, King Faisal University, Alahsa, Saudi Arabia. Data were collected between April 2025 and August 2025. The study aimed to assess leadership competencies among medical students and identify demographic and academic factors associated with leadership competence.

### 2.2 Sampling strategy and sample size determination

A proportionate stratified sampling technique was used to achieve adequate representation of students across all academic levels. Each academic year (Year 1 to Year 5) was treated as an independent stratum. The total study population consisted of 1,617 medical students distributed across the five academic years: Year 1 (*n* = 360), Year 2 (*n* = 376), Year 3 (*n* = 333), Year 4 (*n* = 273), and Year 5 (*n* = 275) (Figure 1).

**Figure 1:**
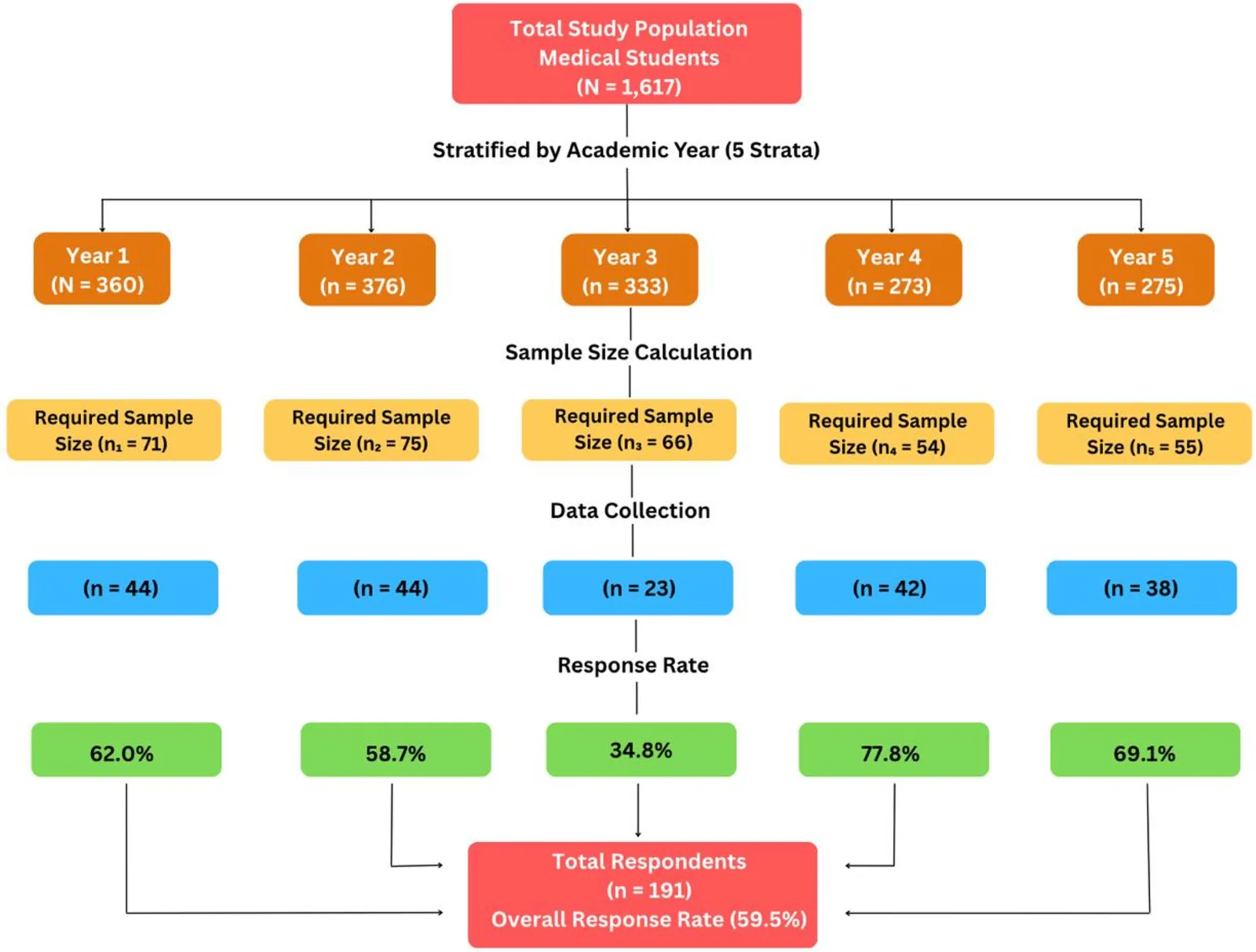
Stratified sampling and participant recruitment process.

The required overall sample size was calculated using the standard formula for finite populations:

$$n\;=\;N\;/\;[1\;+\;N(e^{2})]$$

where *n* represents the required sample size, N represents the total population size, and e represents the margin of error. Assuming a 95% confidence level and a 5% margin of error (e = 0.05), the calculated minimum sample size was 321 participants.

To maintain proportional representation of each academic year, proportional stratified allocation was applied using the formula:

$$n_{h}\;=\;(N_{h}\;/\;N)\;\times \;n$$

where nₕ represents the sample size required from stratum h, Nₕ represents the population size of that stratum, N represents the total population, and *n* represents the overall sample size. Based on this allocation, the required sample sizes were 71 students from Year 1, 75 from Year 2, 66 from Year 3, 54 from Year 4, and 55 from Year 5.

During the data collection period, a total of 191 students completed the survey, yielding an overall response rate of 59.5%. The number of respondents from each academic year was as follows: Year 1 (*n* = 44), Year 2 (*n* = 44), Year 3 (*n* = 23), Year 4 (*n* = 42), and Year 5 (*n* = 38), corresponding to response rates of 62.0%, 58.7%, 34.8%, 77.8%, and 69.1%, respectively.

Because the achieved sample (*n* = 191) was smaller than the calculated minimum sample size (*n* = 321) and response rates differed across academic years, the final sample did not fully preserve the planned proportional allocation. In particular, Year 3 students constituted 12.0% of the sample compared with 20.6% of the student population, whereas Year 4 students constituted 22.0% of the sample compared with 16.9% of the population. The implications of this are considered in the Limitations section.

### 2.3 Data collection instrument

Data were collected using a structured self-administered questionnaire consisting of two sections. The first section included demographic and academic characteristics of the participants, such as age, gender, academic year, and grade point average (GPA). Additional variables related to leadership exposure were also collected, including previous leadership training, participation in leadership workshops, experience holding leadership roles, and aspirations to pursue leadership positions in healthcare.

The second section consisted of the Medical Leadership Competency Scale (MeLeCoS) (17), a validated instrument developed to assess leadership competence among medical students. The MeLeCoS scale includes 37 items distributed across six leadership domains: self-management, responsible behavior, working in teams, managing services, innovation in undergraduate education, and systemic perspectives (17). Responses were recorded using a Likert-type scale reflecting participants’ perceived leadership competence.

### 2.4 Data collection procedure

The questionnaire was distributed electronically to selected students using an online survey platform. Participation was voluntary, and students completed the questionnaire anonymously. The self-administered format allowed participants to respond independently without investigator influence.

### 2.5 Statistical analysis

Regarding the statistical analysis, it was performed via IBM SPSS Statistics 28.0. Using Cronbach's alpha coefficient, the internal consistency of the 37-item Medical Leadership Competency Scale (MeLeCoS) was evaluated. Qualitative data were presented in the form of descriptive statistics, which included frequency, percentages, mean with standard deviation (SD) along with other measures like range and median. The distributional characteristics i.e., skewness and kurtosis were calculated to check normality of data, for the global leadership score and its six subscales. In addition, independent sample t-tests were applied for binary categorical variables like gender and career aspiration, to identify differences in the leadership competence across various demographic groups. A one-way Analysis of Variance was applied for comparisons across the five years of study. The assumptions of homogeneity of variance were evaluated using Levene's test and wherever the assumptions of homogeneity of variances were violated, the Welch robust test of equality (Welch's ANOVA) was used, followed by Games-Howell *post-hoc* comparison. Moreover, Pearson's correlation coefficients (r) were used to assess relationships between various continuous variables, for example, age, GPA, and leadership scores, etc. Last but not the least, a multiple linear regression analysis was employed to identify unique predictors of leadership competence. To ensure model stability, collinearity diagnostics (Variance Inflation Factor i.e., VIF and Condition Index) were employed. A *p*-value of less than 0.05 was considered statistically significant for all the tests.

## 3 Results

The study population included 191 medical students with the values for mean age and mean GPA as follows: 21.76 (1.53) and 4.32 (0.63) respectively. Regarding gender distribution, females accounted for 59.7% of the total study population. The sample was distributed across both the pre-clinical (Years 1–3) and clinical years (Years 4–5) of education. As per the analysis, only 10.5% of participants had received formal leadership training and 71.2% aspired to hold healthcare leadership roles in the future. The Cronbach's Alpha of the 37-item MeLeCoS scale was evaluated to be 0.924 demonstrating an excellent internal consistency.

The overall mean leadership score was calculated to be 3.72 ± 0.58. A normality test was applied to the data, the results of which proved that the data was normally distributed (skewness: −0.217; kurtosis: −0.134; mean = 3.72; median = 3.73), making it suitable for further parametric testing. The detailed demographic and academic characteristics of the study population are present in Table 1.

**Table 1:** Demographic and academic characteristics of the participants.

Variable	Category	Frequency (n)	Percentage (%)
Age (years)	Mean ± SD	21.76 ± 1.53	-
	Range	19–25	-
GPA (out of 5)	Mean ± SD	4.32 ± 0.63	-
Gender	Male	77	40.3
	Female	114	59.7
Year of Study	Year 1 (Pre-clinical)	44	23.0
	Year 2 (Pre-clinical)	44	23.0
	Year 3 (Pre-clinical)	23	12.0
	Year 4 (Clinical)	42	22.0
	Year 5 (Clinical)	38	19.9
Leadership competence score	Mean score ± SD	3.72 ± 0.58	-
	Median	3.73	-
	Range	2.16–4.92	-
	Distribution	Normal Distribution(skewness −0.217)	-
Leadership Exposure and Aspirations			
Formal Leadership Training	Yes	20	10.5
	No	171	89.5
Held Leadership Role	Yes	83	43.5
	No	108	56.5
Leadership Workshop Participation	Yes	59	30.9
	No	132	69.1
Aspires Healthcare Leadership	Yes	136	71.2
	No	55	28.8

The data was divided into five Batches, with the frequencies as follows: Batch 220: 38(19.9%), Batch 221; 42(22.0), Batch 222: 23(12.0%), Batch 223: 44(23.0%) and Batch 224: 44(23.0%). Regarding intended medical specialties as reported by the participants, General Surgery (*n* = 19) was the most frequently intended medical specialty, followed by Family Medicine (*n* = 17), Neurology and Obstetrics & Gynecology (*n* = 16). Cardiology, Hematology, Forensic Medicine, ICU and Urology were the least cited specialties. Detailed illustration is present in Figure 2.

**Figure 2:**
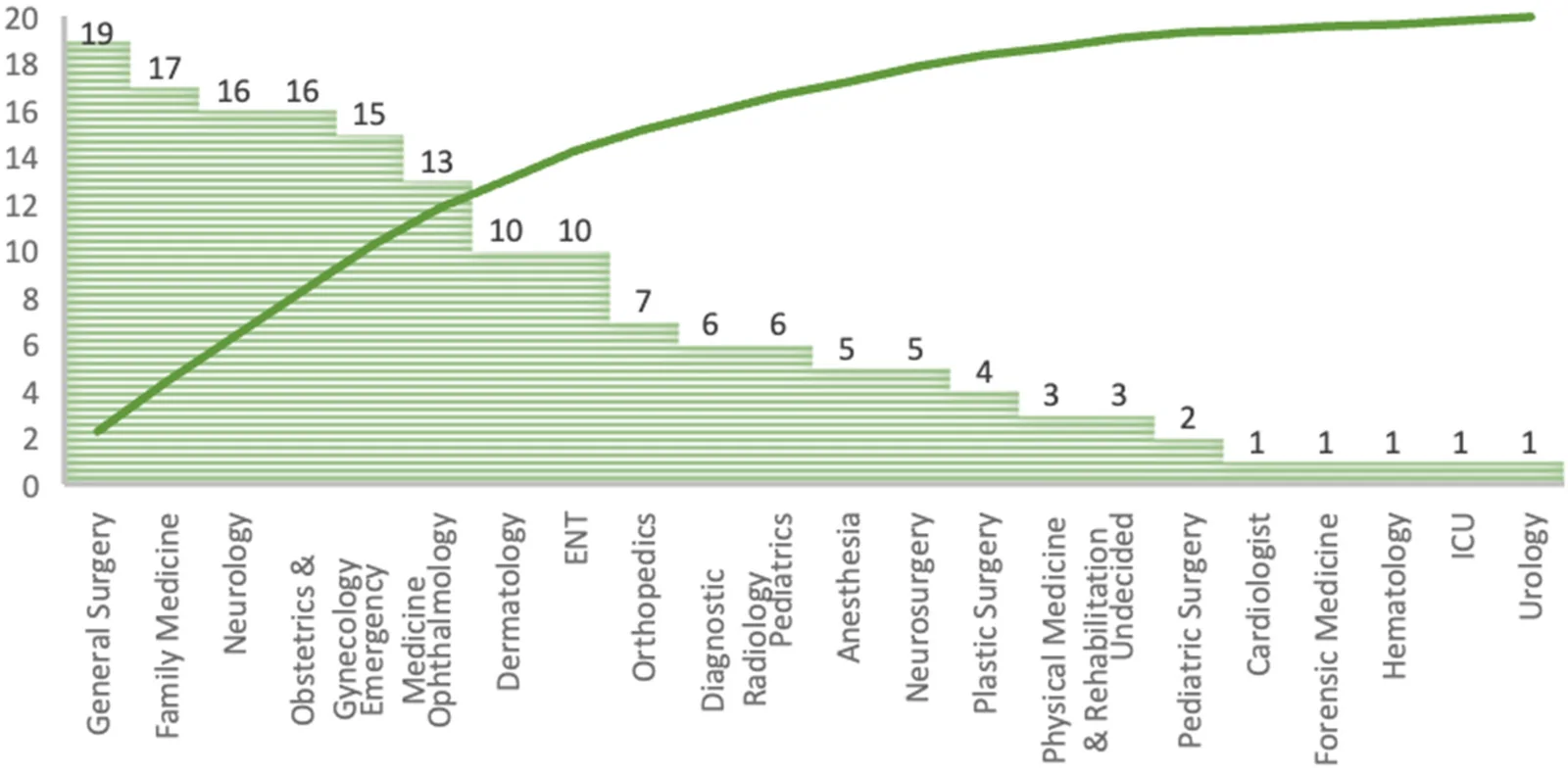
Intended medical specialty.

The Medical Leadership Competency Scale (MeLeCoS) comprises the following six subscales: Factor 1: Self-Management, Factor 2: Responsible behavior, Factor 3: Working in Teams, Factor 4: Managing Services, Factor 5: Innovation in UGE, and Factor 6: Systemic Perspectives (17). Analysis of these subscales yielded some interesting findings indicating a hierarchical perception of competence. Students perceived themselves the most competent in Factor 2, followed by Factor 3 and the least in Factor 5. The reliability coefficients of subscales ranged from 0.788 to 0.845, whereas the item-total correlation had a range of 0.165 to 0.664. Detailed subscale statistics, with skewness and kurtosis, are present in Table 2.

**Table 2:** Descriptive statistics and distributional characteristics of MeLeCoS subscales (*N* = 191).

MeLeCoS Subscale	Mean	SD	Median	Mode	Skewness	Kurtosis	Range	Cronbach's Alpha
F1: Self-Management	3.96	0.59	4.00	3.80	−0.25	−0.45	2.60	0.812
F2: Responsible behavior	4.25	0.74	4.50	5.00	−1.27	1.11	3.33	0.845
F3: Working in Teams	4.01	0.68	4.13	3.88	−0.67	0.01	2.88	0.821
F4: Managing Services	3.61	0.78	3.80	3.80	−0.31	−0.09	4.00	0.788
F5: Innovation in UGE	3.12	0.88	3.13	3.00	0.20	−0.59	3.63	0.804
F6: Systemic Perspectives	3.46	0.75	3.40	3.40	−0.15	−0.29	3.60	0.792
Total	3.72	0.58	3.73	3.51	−0.22	−0.13	2.84	0.924

Among the surveyed specialties, the highest leadership scores were observed in Plastic Surgery (4.30 ± 0.61), followed by Forensic Medicine (4.14 ± 0.5) and Neurosurgery (mean 3.97 ± 0.59). In contrast, the lowest leadership scores were reported in ICU (2.86 ± 0.5), Orthopedics (mean 3.13 ± 0.54), and Cardiology (mean 3.22 ± SD 0.64). A one-way ANOVA showed no statistically significant association between specialty choice and leadership score. (*p* = 0.337).

Bivariate analyses, including independent samples t-tests, one-way ANOVA, Welch's ANOVA (where variance assumptions were violated), and correlation analysis were performed to understand significant associations between study variables. As per t-test, female students were found to have significantly higher global competence than their male colleagues (t(189) = 3.42; *p* = 0.001). In addition, students having leadership aspirations outscored their fellows with a statistically significant difference (t(189) = 3.97; *p* < 0.001). To check association between seniority and leadership competence scores, a Welch's ANOVA was applied which indicated statistically significant differences in the leadership competence subscale Factor 2 (Responsible behavior) by the year of study (F = 3.86; *p* = 0.006). These associations were further confirmed by Games-Howell *post-hoc* tests, according to which, the Year 5 students (Clinical) had significantly greater amount of confidence in their ethical responsibilities (M = 4.56) when compared to Pre-clinical students belonging to Year 1 (*p* = 0.028) or Year 2 (*p* = 0.021). Academic performance (GPA) was found to have a significant positive correlation (r = 0.217; *p* = 0.006). Surprisingly, age yielded no statistically significant association with leadership competence (r = 0.100; *p* = 0.169). The major findings are summarized in Table 3.

**Table 3:** Bivariate analyses for global leadership competence (*N* = 191).

Independent Variable	Test Statistic	Value	df	*p*-value	Result
Gender	Independent t-test	3.42	189	0.001	Significant
Career Aspiration	Independent t-test	3.97	189	<0.001	Significant
Year of Study	Welch's F	3.86	4, 84.3	0.006	Significant^a^
Academic Performance	Pearson's correlation	0.217	160	0.006	Significant
Chronological Age	Pearson's correlation	0.100	188	0.169	Non-Significant

a For Year of Study, significance refers specifically to Factor 2 (Responsible behavior). Global mean differences were non-significant (*p* = 0.336).

To check the potential predictors affecting student leadership scores, a multiple linear regression analysis was applied, according to which a 10.6% variance was observed in leadership scores (F(4, 157)= 4.69; *p* = 0.001) i.e., statistically significant. When adjusting for all other variables, two significant predictors were observed: gender (*β* = 0.256, *p* = 0.002) and GPA (*β* = 0.201, *p* = 0.010). These findings suggest that gender and academic performance were significant predictors of leadership competence in this model, whereas seniority and marital status were not. Nevertheless, the model accounted for only a modest proportion of the variance in leadership competence (R^2^ = 0.106). The details of multiple linear regression analysis, along with beta and *p*-values are given in Table 4. Collinearity diagnostics reported a Condition Index of 23.0 (Max VIF=1.1) which confirmed the stability of the model.

**Table 4:** Multiple linear regression analysis for global leadership competence.

Predictor	B	SE	Beta (*β*)	t	Sig. (p)
(Constant)	−0.03	1.48	-	−0.02	0.984
Gender (Female)	0.29	0.09	0.256	3.22	0.002*
GPA	0.18	0.07	0.201	2.62	0.010*
Year of Study	−0.10	0.08	−0.270	−1.30	0.195
Marital Status	−0.19	0.17	−0.088	−1.13	0.262

R2 = 0.106; *p < .05.

## 4 Discussion

### 4.1 Current literature

Leadership competence should be cultivated during undergraduate medical education, given that physicians frequently engage in leadership-related responsibilities as part of their daily clinical practice (5, 8, 18). A growing body of evidence has demonstrated that leadership and management play crucial roles in delivering high-quality patient care, with effective leadership styles being associated with improved patient safety, lower mortality rates, and greater patient satisfaction across different healthcare settings (19, 20). In response to these demands, medical education systems worldwide have increasingly emphasized leadership development as part of competency-based training frameworks, and many institutions have begun integrating leadership curricula, interprofessional education, and team-based learning approaches to better prepare future physicians for leadership responsibilities within complex healthcare systems (8, 18, 21). Nevertheless, although leadership and management competencies are increasingly recognized as important components of medical training, several scholars have argued that leadership development should begin early in undergraduate medical education rather than being deferred to postgraduate training, in order to avoid what has been described as “accidental leadership,” whereby physicians are expected to assume leadership responsibilities without prior formal preparation (5). Despite this growing recognition, much of the existing literature has focused on students’ attitudes toward leadership or their interest in leadership roles, while fewer studies have examined self-perceived leadership competence using structured multidimensional assessment tools such as the Medical Leadership Competency Scale (MeLeCoS) (12, 17, 22–24). Therefore, this study was conducted to examine self-perceived leadership competence among medical students in Saudi Arabia, to better understand how leadership skills develop during undergraduate medical training, and to identify potential gaps in current educational approaches.

### 4.2 Inspiration and motivation toward leadership positions

One of the notable findings of this study was the discrepancy between students’ leadership aspirations and their exposure to formal leadership education. Although only 10.5% of participants reported prior formal leadership training, 71.2% expressed interest in assuming future leadership roles within healthcare. This pattern suggests that leadership development is not yet systematically embedded within undergraduate medical education, despite substantial student interest. Limited formal exposure may reflect competing curricular demands and inconsistent integration of leadership training within existing programs. In our setting, the relatively high level of leadership interest may also be partly explained by students’ engagement in extracurricular activities and the availability of student-led structures within the college, such as the medical student council, which may make leadership exposure more accessible even outside the formal curriculum. This interpretation is supported by previous work showing that participation in extracurricular activities among medical students is often motivated by shaping personality and developing transferable skills, alongside more strategic aims such as strengthening the curriculum vitae (25).

This interpretation is consistent with prior literature. In a multi-institutional UK study, only 7.9% of final-year medical students felt well informed about leadership and management roles in healthcare practice, whereas 63.2% reported that they would have liked more leadership training during medical school, 65.8% expressed interest in taking on future leadership or managerial responsibilities, and 78% believed such opportunities should be more clearly promoted to students (23). More than half of respondents in that study also rated the leadership and management training they had received as poor (23). Similar findings were reported in Saudi Arabia, where 43.2% of students wanted more leadership training during medical school, 38.4% were willing to seek additional leadership or management training during postgraduate study, and 73.2% agreed that leadership opportunities should be highlighted and promoted to students (12). Together, these findings suggest that the gap observed in our cohort reflects a broader pattern in which student interest in leadership exceeds the structure of formal undergraduate provision (12, 23).

Previous research examining leadership training initiatives has shown that educational workshops incorporating communication skills and emotional intelligence are generally well received by medical students. Participants frequently describe these experiences as enjoyable, engaging, and valuable, which may help explain the strong interest in leadership roles observed among medical students and underscores the importance of integrating structured leadership development opportunities within undergraduate medical education (26).

These findings may also be considered alongside our gender-related results. In the present study, female students demonstrated significantly higher leadership competence scores. However, prior work has shown that male medical undergraduates may be more likely to strongly identify themselves with future leadership positions. In a pilot study, male students were more likely than female students to strongly agree that they saw themselves in a future leadership role, while lack of confidence, tradition, and perceived barriers to acceptance were identified as obstacles to women's leadership progression (24). Taken together, these findings may suggest that leadership competence and future leadership self-positioning are not necessarily equivalent constructs, and may be shaped by different educational and social influences (24). The same study also highlighted mentoring, role models, and greater curricular attention to leadership development at the undergraduate level as important supports for future leadership development (24). Overall, our findings suggest that medical students appear willing to engage with leadership development, but that this willingness is not yet matched by equivalent formal training exposure during undergraduate medical education.

### 4.3 Leadership competence scores

The findings of this study demonstrated an overall leadership competence score above the scale midpoint among medical students, with a mean MeLeCoS score of 3.72 ± 0.58. At the subscale level, the highest scores were observed in Factor 2 (Responsible behavior), Factor 3 (Working in teams), and Factor 1 (Self-management), whereas the lowest scores were observed in Factor 5 (Innovation in undergraduate medical education), Factor 6 (Systemic perspectives), and Factor 4 (Managing services), respectively.

These findings are broadly consistent with prior MeLeCoS-based research. In the development and validation study of the MeLeCoS, Factor 2 (“Demonstrating responsible behaviour and shaping relations”) showed the highest mean score, whereas Factor 5 (“Promoting improvement and innovation in undergraduate medical education”) showed the lowest (17). The authors further explained that the six MeLeCoS factors could be grouped into three broader domains: competencies related to the student as an individual leader (Factors 1 and 2), competencies related to the student's interaction with healthcare organizations and management processes (Factors 3 and 4), and competencies related to leadership within undergraduate medical education and the wider healthcare system (Factors 5 and 6) (17).

Within this framework, the pattern observed in our study suggests that students felt more confident in domains closer to their individual and interpersonal experiences, while reporting lower confidence in domains related to educational innovation, service management, and broader system-level perspectives (17). This interpretation is also supported by the MeLeCoS development study, in which the lowest-scoring items were mainly related to students’ active involvement in councils, committees, and projects aimed at improving undergraduate medical education (17). This may reflect the fact that opportunities to participate in educational improvement and institutional change are usually available only to a limited number of students and are not part of the routine experience of most undergraduate medical students (17).

A similar conclusion was suggested by the follow-up MeLeCoS study conducted during the final year of medical education. Although the authors hypothesized that immersion in clinical environments and exposure to physician role models would lead to measurable improvements in leadership competence, no significant differences were found either in the overall subscale scores or in the individual questionnaire items (22). This finding suggests that passive clinical exposure alone may be insufficient for meaningful leadership development and supports the need for structured, longitudinal leadership training within undergraduate medical curricula (22).

This interpretation is further reinforced by recent curriculum-development work. Nicolaou et al. argued that leadership and management training should begin early in medical school, and their pilot module, delivered through five 2-hour online sessions, resulted in increased self-reported competence in 18 of 22 leadership-related skills after participation (27). Taken together, these findings suggest that competencies related to management, innovation, and systems thinking may be less likely to develop through routine undergraduate exposure alone and may instead require intentional curricular support (27).

### 4.4 Contribution in relation to existing competency frameworks

Applying the multidimensional MeLeCoS framework in this setting adds information that is not available from previous Saudi studies of attitudes and training needs (12–14). Rather than indicating only whether students value leadership training, it provides a domain-level profile of where students perceive themselves to be more or less competent. The six MeLeCoS factors conceptually overlap with the MLCF domains of personal qualities, working with others, managing services, improving services, and setting direction (9, 17). Our findings suggest that, at the undergraduate level, these domains are not perceived to develop evenly: students rated themselves highest in the personal and interpersonal domains (responsible behavior, working in teams, and self-management) and lowest in the domains related to managing services, innovation, and systemic perspectives. This pattern may refine the application of the MLCF to undergraduate education by indicating that the organizational and system-level domains may require deliberate curricular attention rather than being expected to emerge from routine training (9, 22, 27).

In the Saudi context, SaudiMEDs and its comparison with CanMEDs emphasize competency domains such as professionalism, communication, and teamwork (10, 11). Our results suggest that students perceive themselves as relatively competent in these areas, whereas the service-management, improvement, and system-level aspects of leadership appear less developed. These findings may therefore help curriculum planners identify which leadership domains need more explicit learning outcomes, learning activities, and assessment within SaudiMEDs-aligned undergraduate curricula. Because these data are cross-sectional and based on self-assessment, they should be regarded as hypothesis-generating and should be confirmed using longitudinal designs and observed or multi-source measures of leadership performance.

### 4.5 Specialty preferences among students

Regarding intended career pathways, general surgery emerged as the most frequently selected specialty, followed by family medicine, neurology, and obstetrics and gynecology. Specialty preference may shape how students perceive future professional roles and leadership responsibilities within healthcare systems.

Our findings can be viewed alongside previous Saudi data. Previous Saudi research demonstrated that students preferring medical specialties had higher leadership-related perception scores than those preferring surgical specialties (14). In contrast, general surgery was the most frequently selected specialty in our cohort, and one-way ANOVA showed no statistically significant association between specialty preference and leadership score overall.

Descriptively, the highest mean leadership scores in our cohort were observed among students interested in plastic surgery, forensic medicine, and neurosurgery, whereas the lowest mean scores were observed among those interested in ICU, orthopaedics, and cardiology. However, these subgroup differences should be interpreted cautiously, as several specialty categories were represented by small numbers of students.

The relatively high interest in surgical specialties in our cohort may nevertheless partly reflect the leadership demands inherent to surgical practice. Surgical work commonly requires coordination within multidisciplinary teams, effective communication, and timely decision-making in high-pressure clinical environments, particularly in the operating room and perioperative setting (28, 29). These features may help explain why surgical careers are perceived by some students as leadership-intensive clinical pathways.

### 4.6 Predictors of leadership competence

The present study identified several factors associated with leadership competence among medical students. Female students demonstrated significantly higher leadership competence scores compared with male students, and students who expressed aspirations toward leadership roles also achieved higher competence scores. In addition, seniority was associated with differences in specific leadership domains, with final-year students demonstrating greater confidence in ethical responsibilities compared with junior students, suggesting progressive development in responsibility-related competencies during clinical training. Although age was not significantly associated with leadership competence, this finding suggests that chronological maturity alone may not explain variation in leadership scores (22).

Academic performance was positively associated with leadership competence (r = 0.217, *p* = 0.006), indicating that students with stronger academic achievement may also exhibit higher levels of self-management, discipline, and professional engagement, which are integral components of leadership development. In contrast, age showed no significant association, further reinforcing the concept that experiential learning plays an important role in leadership development (22, 27).

Multiple linear regression analysis demonstrated that the model significantly predicted leadership competence, explaining approximately 10.6% of the variance in leadership scores. Within this model, gender and GPA emerged as the only significant predictors, whereas seniority and marital status were not statistically significant. Although the explained variance was modest, this finding highlights that leadership competence is a multifactorial construct influenced by a complex interplay of academic, behavioral, and contextual factors beyond those captured in the current model. This may also reflect the role of the hidden curriculum in medical education, where informal learning experiences, role modeling, and the clinical environment contribute substantially to leadership development beyond formal training structures (30). Beyond formal training and academic characteristics, leadership development may also be shaped by context-specific expectations regarding the attributes associated with effective leaders. A qualitative study among managers in a medical sciences university showed that implicit leadership theories encompass multiple personal, interpersonal, managerial, and ethical characteristics, suggesting that leadership perceptions are embedded within the organizational and cultural context in which leadership is enacted (31).

Similar observations have been reported in studies examining predictors of leadership development among healthcare students. For instance, research among Saudi nursing students identified emotional intelligence and self-esteem as important predictors of leadership competency (32). Additionally, leadership behaviors within clinical learning environments may influence students’ professional development. A mixed-methods study investigating feedback-seeking behaviours among medical students reported that supportive leadership styles within clinical teams encouraged students to actively seek feedback and engage more effectively in the learning process (33).

### 4.7 Leadership development initiatives

Leadership development initiatives for university students in Saudi Arabia have expanded considerably in recent years, particularly within the national transformation framework of Vision 2030, which emphasizes the development of future leaders across multiple sectors (34–36).

Programs such as the Qimam Fellowship Program aim to identify high-performing university students and provide them with mentorship opportunities, leadership training, and professional networking experiences (34). Similarly, the Misk Foundation has launched numerous initiatives designed to promote leadership development, innovation, and entrepreneurial skills among Saudi youth (35).

Within the healthcare sector, leadership training has also been incorporated into postgraduate medical education. Saudi Commission for Health Specialties curricula and accreditation standards include leadership- and professional development-related expectations within residency training (36).

Internationally, structured frameworks have also been developed to guide physician leadership development. For example, the Medical Leadership Competency Framework introduced by the United Kingdom's National Health Service outlines leadership competencies expected of physicians throughout medical training and professional practice (9). Such frameworks highlight the increasing recognition of leadership as an essential competency for healthcare professionals worldwide.

Despite these initiatives, structured leadership education remains inconsistently integrated within undergraduate medical curricula. While extracurricular programs provide valuable opportunities for leadership exposure, incorporating formal leadership training into medical education curricula may provide a more systematic approach to preparing future physicians for leadership roles within healthcare systems (22, 27).

### 4.8 Strengths and limitations

This study has several strengths. The use of the validated Medical Leadership Competency Scale (MeLeCoS) provided a structured and reliable tool for assessing leadership competence among medical students. In addition, a proportionate stratified sampling design was used so that students from all five academic years were included in the sample. The inclusion of students from both pre-clinical and clinical phases of training allowed for a broader evaluation of leadership competence across different stages of medical education. Furthermore, the study employed comprehensive statistical analyses, including correlation and regression models, which enabled the identification of demographic and academic predictors associated with leadership competence.

However, several limitations should also be acknowledged. The cross-sectional design of the study limits the ability to infer causal relationships between the studied variables and leadership competence. In addition, data were collected using a self-administered questionnaire, which may introduce response bias or social desirability bias, as participants may overestimate their leadership abilities. The achieved sample (*n* = 191; overall response rate 59.5%) was smaller than the calculated minimum sample size of 321, which reduces the precision of our estimates; with this sample, the margin of error at the 95% confidence level is approximately 6.7% rather than the planned 5%. Stratum-specific response rates also varied considerably, from 34.8% in Year 3 to 77.8% in Year 4, so that Year 3 students were under-represented and Year 4 students were over-represented relative to the student population. Non-response bias cannot be excluded, as students with greater interest in leadership may have been more likely to participate; the findings may therefore not be fully representative of all medical students at the college. The study was conducted within a single medical institution, which may limit the generalizability of the findings to other medical schools in Saudi Arabia or internationally. Finally, the MeLeCoS scale measures self-perceived leadership competence and does not directly assess actual leadership performance in real clinical or healthcare settings; our findings should therefore be interpreted as students’ perceived competence rather than as observed leadership ability.

## 5 Conclusion

This study assessed self-perceived leadership competence among medical students at King Faisal University using the Medical Leadership Competency Scale (MeLeCoS). Overall, students demonstrated a moderate to high level of perceived leadership competence, with the highest scores observed in responsible behavior and teamwork, while innovation in undergraduate education showed comparatively lower scores. Female students and those with higher academic performance exhibited significantly higher self-perceived leadership competence scores, suggesting that both demographic and academic factors may influence leadership development during medical training.

These findings highlight the importance of integrating structured leadership development initiatives within undergraduate medical curricula. Providing formal leadership training, mentorship opportunities, and leadership-focused educational activities may further strengthen leadership competencies among medical students and better prepare future physicians to lead within complex healthcare systems.

## Data Availability

The datasets presented in this article are not publicly available due to patient confidentiality. The data will be made available upon reasonable request from the corresponding author.
